# Penetrance and expressivity of mitochondrial variants in a large clinically unselected population

**DOI:** 10.1093/hmg/ddad194

**Published:** 2023-11-21

**Authors:** Stuart J Cannon, Timothy Hall, Gareth Hawkes, Kevin Colclough, Roisin M Boggan, Caroline F Wright, Sarah J Pickett, Andrew T Hattersley, Michael N Weedon, Kashyap A Patel

**Affiliations:** Department of Clinical and Biomedical Sciences, University of Exeter, 79 Heavitree Road, Exeter, EX2 4TH, United Kingdom; Department of Clinical and Biomedical Sciences, University of Exeter, 79 Heavitree Road, Exeter, EX2 4TH, United Kingdom; Department of Clinical and Biomedical Sciences, University of Exeter, 79 Heavitree Road, Exeter, EX2 4TH, United Kingdom; Exeter Genomics Laboratory, RILD Building, Royal Devon University Healthcare NHS Foundation Trust, Barrack Road, Exeter, EX2 5DW, United Kingdom; Wellcome Centre for Mitochondrial Research, Translational and Clinical Research Institute, Newcastle University, Framlington Place, Newcastle upon Tyne, NE2 4HH, United Kingdom; Department of Clinical and Biomedical Sciences, University of Exeter, 79 Heavitree Road, Exeter, EX2 4TH, United Kingdom; Wellcome Centre for Mitochondrial Research, Translational and Clinical Research Institute, Newcastle University, Framlington Place, Newcastle upon Tyne, NE2 4HH, United Kingdom; Department of Clinical and Biomedical Sciences, University of Exeter, 79 Heavitree Road, Exeter, EX2 4TH, United Kingdom; Department of Clinical and Biomedical Sciences, University of Exeter, 79 Heavitree Road, Exeter, EX2 4TH, United Kingdom; Department of Clinical and Biomedical Sciences, University of Exeter, 79 Heavitree Road, Exeter, EX2 4TH, United Kingdom

**Keywords:** Mitochondria, UK biobank, Mitochondrial disease, Maternally inherited diabetes and deafness

## Abstract

Whole genome sequencing (WGS) from large clinically unselected cohorts provides a unique opportunity to assess the penetrance and expressivity of rare and/or known pathogenic mitochondrial variants in population. Using WGS from 179 862 clinically unselected individuals from the UK Biobank, we performed extensive single and rare variant aggregation association analyses of 15 881 mtDNA variants and 73 known pathogenic variants with 15 mitochondrial disease-relevant phenotypes. We identified 12 homoplasmic and one heteroplasmic variant (m.3243A>G) with genome-wide significant associations in our clinically unselected cohort. Heteroplasmic m.3243A>G (MAF = 0.0002, a known pathogenic variant) was associated with diabetes, deafness and heart failure and 12 homoplasmic variants increased aspartate aminotransferase levels including three low-frequency variants (MAF ~0.002 and beta~0.3 SD). Most pathogenic mitochondrial disease variants (n = 66/74) were rare in the population (<1:9000). Aggregated or single variant analysis of pathogenic variants showed low penetrance in unselected settings for the relevant phenotypes, except m.3243A>G. Multi-system disease risk and penetrance of diabetes, deafness and heart failure greatly increased with m.3243A>G level ≥ 10%. The odds ratio of these traits increased from 5.61, 12.3 and 10.1 to 25.1, 55.0 and 39.5, respectively. Diabetes risk with m.3243A>G was further influenced by type 2 diabetes genetic risk. Our study of mitochondrial variation in a large-unselected population identified novel associations and demonstrated that pathogenic mitochondrial variants have lower penetrance in clinically unselected settings. m.3243A>G was an exception at higher heteroplasmy showing a significant impact on health making it a good candidate for incidental reporting.

## Introduction

Mitochondrial function is fundamental to human life but can be impaired by pathogenic mitochondrial (mt)DNA variants, leading to disease with variable expressivity and penetrance [1–3]. Studies of predominantly clinically ascertained cohorts have identified > 90 pathogenic variants in the mitochondrial genome [1, 2, 4, 5]. These variants cause rare heterogeneous mitochondrial disorders, including complex multi-organ disease [1]. Multiple studies have also reported mtDNA variants associated with complex diseases such as maternally-inherited diabetes [6, 7], metabolic diseases [8, 9], Parkinson's disease [10], neuroticism [11], as well as stroke and psoriasis [12], among others. These variants provide insight into the function of human mitochondria and the pathogenesis underlying these diseases [13].

Previous mitochondrial DNA studies have had limitations. For example, when identifying mitochondrial variants which affect complex traits, studies have either assessed a subset of common variants (n ~ 700) in large, unselected cohorts [14, 15], or all variants in small, often clinically selected cohorts (n = 100–2800) focussing on a certain phenotype [16, 17]. Studies of pathogenic variants have been primarily restricted to patients and family members with specific phenotypes, likely resulting in over-inflated penetrance and expressivity estimates, as seen for nuclear pathogenic variants [18–20]. Large-scale rare and common mitochondrial genome-wide association studies in clinically unselected populations can overcome these limitations, and lead to novel insights into the impact of mitochondrial variants on human health [21].

The recent availability of whole genome sequencing data (WGS) from large cohorts provides a unique opportunity to study rare and common mitochondrial variants. WGS data have recently been made available in large population cohorts such as the UK Biobank (UKB, n = 200 030) [22] and All of Us [23]. WGS captures information from both mitochondrial DNA and nuclear DNA, providing high-quality genotyping for all mitochondrial variations, both rare and common. Importantly, it also provides accurate data on variant heteroplasmy (proportion of mtDNA molecules with alternate allele) and mtDNA copy number (mtCN), making it an ideal technology to study all, and particularly rare, heteroplasmic and homoplasmic mitochondrial variation [24]. These large cohort studies also provide excellent opportunity to assess penetrance and expressivity of known pathogenic variants in a clinically unselected population which is crucial before considering the incidental reporting of mitochondrial variants.

Here we use WGS data from a large multi-ethnic population cohort of 200 030 individuals from the UKB. We aimed to identify novel rare and common variants associated with 15 common mitochondrial disease-related traits and assess the prevalence and penetrance of known pathogenic variants in an unselected population cohort.

## Results

### Mitochondrial-wide single variant association identified associations with diabetes, deafness, heart failure and aspartate aminotransferase

**Figure 1:**
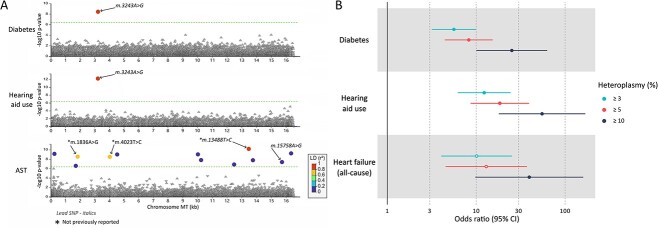
Summary of hypothesis free mitochondrial genome wide associations for 15 mitochondrial disease phenotypes and *m.3243A>G* associations at increasing heteroplasmy thresholds. (A) LocusZoom plot highlighting all significant associations between mitochondrial variants in the UK biobank with measured heteroplasmy ≥ 3% (n = 8896, MAC ≥ 5) and the 15 tested phenotypes. Dashed line denotes the Bonferroni-corrected significance threshold (*P* < 4.03×10^−7^). R^2^ correlation is relative to the most significant variant in each plot. (B) Forest plot of *m.3243A>G* associations at increasing minimum heteroplasmy. Solid points denote associations passing the Bonferroni significance threshold (*P* < 3.75×10^−7^). Heart failure (all-cause) was the only phenotype to have a significant association in the sensitivity tests for any traits. AST = aspartate transferase.

We performed single variant association analyses between 8896 mitochondrial variants with a minor allele count (MAC) ≥ 5 and 15 disease/traits that are commonly reported in individuals with mitochondrial disease (Tables S1 and S2). In a European-like ancestry analysis, we identified four disorder/traits where variants were associated at our Bonferroni-corrected significance threshold (*P* < 3.75×10^**−7**^) of which enzyme aspartate aminotransferase (AST) was the only trait with > 1 variant association. This included 12 homoplasmic variants associated with AST and only one heteroplasmic variant (m.3243A>G) associated with diabetes (MAF = 0.00024, OR = 5.6, 95% CI [3.2–9.9], P = 4×10^−9^) and hearing aid use (OR = 12.3, 95% CI [6.2–24.4], P = 6×10^−13^  Fig. 1A, Table S3). Sensitivity analysis using a higher heteroplasmy threshold (≥ 10%) observed one additional association of heart failure with the same m.3243A>G variant (OR = 39.5, 95% CI [9.76–160.1]; *P* < 3×10^−7^; Table S1). Sensitivity analysis using the dominant model of association (reference vs either heteroplasmic or homoplasmic) did not identify additional variants and showed consistent results.

### Lead AST associated variant associates with other liver enzymes but not non-alcoholic fatty liver disease

Of the 12 primarily homoplasmic variants associated with increased AST levels, three were low frequency novel variants with a large effect size (MAF ~ 0.002, β ~ 0.33) compared to previously known variants (Fig. 1, Tables S1, S3 and S4). The conditional analysis of each variant for all others showed that the AST associations were led by one previously reported variant: m.15758A>G (MAF = 0.02; β = 0.09, P = 5×10^−8^) [14] and one novel variant: m.13488C>T (MAF = 0.002; β = 0.33, P = 7×10^−11^) (Table S3). m.15758A>G causes a missense change in the MY-CYB gene and m.13488C>T is a synonymous change in the MT-ND5, both components of respiratory chain complexes. We next tested the association of these two lead variants with four other liver biomarkers; alanine aminotransferase (ALT), alkaline phosphatase (ALP), gamma-glutamyl transferase (GGT), bilirubin and, non-alcoholic fatty liver disease (NAFLD). Both these variants associated with increased ALT (beta 0.06 and 0.24 respectively, *P* < 6×10^−4^) but neither were associated with any other liver markers or non-alcoholic fatty liver disease (Table S5).

### Rare variant aggregation and other ancestry analyses did not identify additional novel associations

We next performed rare variant (MAF < 0.001) aggregation burden analyses to determine whether rare mtDNA variants were associated with these mitochondrial-related traits. We aggregated rare variants by mitochondrial transcript and for 100 bp sliding windows at 10 bp intervals. We did not identify any new associations at a Bonferroni-corrected *P* value (< 2×10^−6^; =0.05/1696/15). We calculated the power to identify a variant associated with diabetes equivalent to m.3243A>G (MAF 0.0002, OR = 5.6) at alpha 1×10^−7^ in the AFR-like and SAS-like individuals which was 5.38×10^−6^ and 1.1×10^−5^ respectively. Conversely, to achieve a power of 0.8, we expect to require N = 157 000 and N = 137 000 respectively, at alpha 1×10^−7^, or N = 32 000 and N = 28 000 at alpha = 0.05 for the AFR and SAS individuals respectively. This in in line with our results, where we observed no statistical signal in those populations at Bonferroni corrected significance threshold (Table S1).

### The m.3243A>G association with diabetes, deafness, and heart failure greatly increases with blood heteroplasmy ≥10%

**Figure 2:**
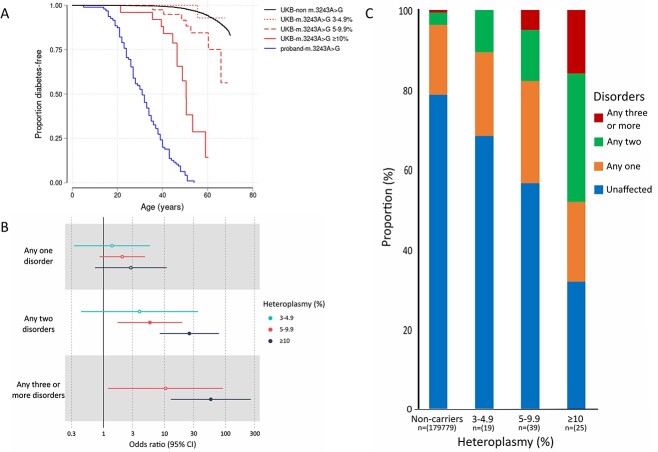
Penetrance of diabetes in *m.3243A>G* carriers in clinical and unselected cohorts and associations with multisystem phenotypic presentations. (A) Kaplan-Meier survival curves of diabetes for m.3243A>G individuals in the UK biobank (n = 83) split by 3%–4.9% (n = 19), 5%–9.9% (n = 39) and ≥ 10% heteroplasmy (n = 25) and clinically identified probands with m.3243A>G (n = 95). The log rank test p value for these three groups against the noncarriers in the UK biobank were 0.8, 5×10^−5^_,_ and 9×10^−71^, respectively. The log rank test *P* value of UKB ≥ 10% group against proband was 1×10^−9^ (B) significant multisystem disorders with one or more presenting phenotypes of: heart failure (all-cause) or non-ischaemic cardiomyopathy, constipation, hearing problems, diabetes, stroke, renal disease, and epilepsy. Hollow points denote non-significant associations when adjusting for multiple testing (*P* < 0.0056; *P* = 0.05/9) (C) proportion of *m.3243A>G* carriers (n = 83) with multiple affected systems at increasing heteroplasmy thresholds split by 3%–4.9% (N = 19), 5%–9.9% (n = 39) and ≥ 10% heteroplasmy (n = 25).

The variant m.3243A>G was associated with diabetes and deafness at ≥ 3% heteroplasmy and these associations were stronger with higher heteroplasmy. The median m.3243A>G heteroplasmy was 7.8% (IQR 5.3–13.1%) for 83 m.3243A>G carriers (all ancestry) in the UKB. Of which, 23% had 3%–4.9%, 47% had 5%–9.9% and 30% had ≥ 10% (Fig. S1). Consistent with cross-sectional analysis, we observed higher age-related penetrance of diabetes using Cox proportional hazard model for diabetes (HR 5.8, 95% CI: [3.8–9], *P* = 3.5×10^−15^) in all m.3243A>G carriers compared to non-carriers (Fig. S2). Although, the overall risk was higher, this was predominantly driven by individuals with heteroplasmy ≥ 5% (Fig. 2A, Table S6). The risk of diabetes was similar to the background/non m.3243A>G carriers for individuals with m.3243A>G heteroplasmy of 3%–4.9% (HR 0.98, 95% CI [0.14–6.9], *P* > 0.9) but increased to 3.8 (95% CI [1.8–8], *P* = 3.9×10^−4^) for 5%–9.9%, and further increased to 20.6 (95% CI [11.7–36.4], *P* = 1.2×10^−25^) for individuals with ≥ 10% (Table S6) (P heterogeneity = 0.0001). Of the 16 386 individuals with diabetes, m.3243A>G was responsible for 0.14% of diabetes. All results were consistent for unrelated European-like ancestry individuals and when using tertiles of age-adjusted m.3243A>G heteroplasmy (based on a previously published formula [25]) (Fig. S3A, Table S7). These data suggest that m.3243A>G level in the blood is an important factor underlying penetrance in a clinically unselected population.

### m.3243A>G is associated with higher risk of multisystem disorder which increases with higher heteroplasmy

The variant m.3243A>G is commonly associated with a multi-system disorder when ascertained clinically. It is not clear if this occurs when assessed in a hypothesis-free manner in clinically unselected cases, and whether any risk is affected by m.3243A>G levels. To assess this, we grouped mitochondrial-related disorders where we observed the nominal association with m.3243A>G at *P* < 0.05 into seven anatomical sites. For example, cardiovascular disorder included non-ischaemic cardiomyopathy all cause heart failure (Table S2). Similar to diabetes, the individuals with 3% to < 5% m.3243A>G levels did not have a statistically significant increased risk of having any one disorder or any two disorders (fisher exact test *P* > 0.1). However the association became significant with increasing heteroplasmy and, compared to non-carriers, individuals with ≥ 10% m.3243A>G heteroplasmy had a higher chance of having any one disorder (OR 2.75, 95% CI [0.71–9.52], *P* = 0.076), increasing to 24.8 (95% CI [8.1–75.7], *P* < 5.1×10^−8^) for any two disorders and 53.50 (95% CI [11.78–199.93], *P* < 3.5×10^−6^) for any three or more disorders (p heterogeneity 0.01) (Fig. 2B). Although the risk of multiple features was high compared to non-carriers, it proportionally affected only a small number of total m.3243A>G carriers (n = 83); 23% (n = 19) with one disorder, 18% (n = 15) with two disorders and 7% (n = 6) with three or more disorders, which increased with higher heteroplasmy (Fig. 2C). This indicates that mutation load is an important factor in the expressivity of these multi-system traits in the unselected population.

### Age-related penetrance of diabetes is lower in unselected populations with m.3243A>G compared to clinically selected diabetes cohorts

Penetrance estimates based on clinically selected probands are often overinflated for nuclear monogenic disorders [20]. However, this ascertainment effect has not been explored in detail for the m.3243A>G variant. We therefore compared the penetrance of diabetes in the UKB to 95 probands with diabetes and m.3243A>G, identified from routine diabetes clinics (Tables S6–S8). The penetrance of diabetes was 96% (95% CI [90–98]) at age 50 in probands, compared to 15% (95% CI 8.5–26) for carriers in the UKB (log rank test *P* = 3×10^−37^) (Fig. S2). The penetrance remained lower at 42% (95% CI [23–69]) even when considering individuals with > 10% heteroplasmy in the UKB compared to proband individuals (*P* = 1×10^−9^). The measured m.3243A>G level was higher in probands compared to UKB (median 7.8 vs 24.6, Fig. S1). Therefore, to assess whether the difference in penetrance is explained by difference in m.3243A>G level, we conducted cox proportional hazard model after adjustment for measured m.3243A>G heteroplasmy, age at recruitment, sex, and body mass index (BMI). We found that risk of diabetes in the UKB for individuals with > 10% heteroplasmy still remained lower compared to probands (adjusted HR 0.55, 95% CI [0.43–0.7], *P* = 9×10^−7^) (Table S8). We observed consistent results using calculated age-adjusted heteroplasmy based on published equation [25] (Table S7). These data suggest that there are additional factors modify the penetrance of diabetes in addition to m.3243A>G levels.

### Type 2 diabetes genetic risk score (T2DGRS) alters penetrance of diabetes with m.3243A>G

**Figure 3:**
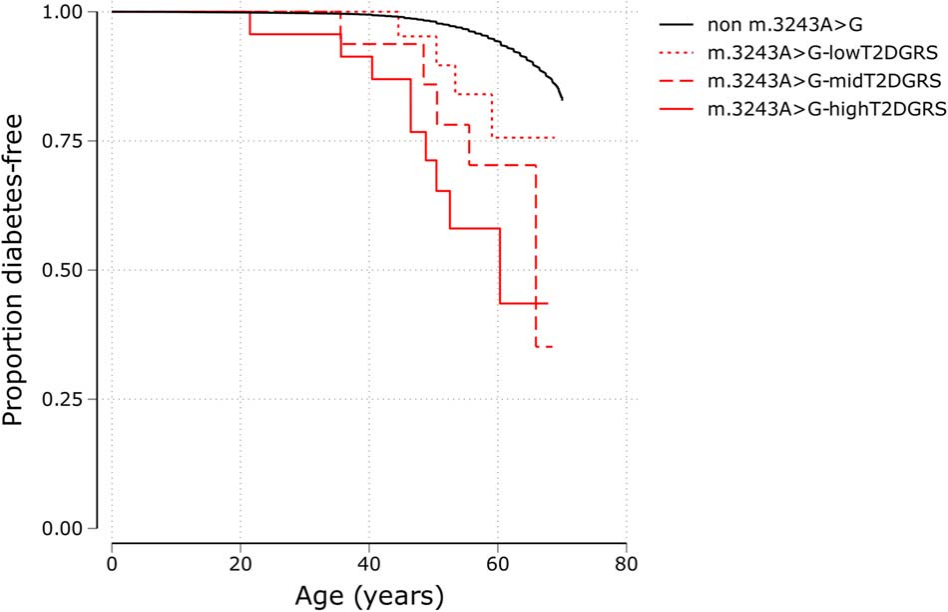
Penetrance of diabetes for individuals with pathogenic *m.3243A>G* variants in an unselected cohort. Kaplan-Meier survival curves of diabetes for m.3243A>G carriers split into tertiles (low, mid, and high) of type 2 diabetes genetic risk score (T2DGRS) and non-carriers (n = 178 340) in the UK biobank. The log rank test p value for low vs mid and low vs high groups was 0.39 and 0.03, respectively.

We hypothesized that nuclear polygenic risk might modify m.3243A>G-related diabetes as seen in nuclear monogenic disorders [26]. We found that the risk of any diabetes at 50 years increased from 5% (95% CI[1–29]) to 14% (95% CI[4–46]) and 29% (95% CI[14–53]) for m.3243A>G carriers for people with low, medium, and high tertiles of T2DGRS, respectively (Fig. 3). This effect was in the same direction after adjusting for m.3243A>G heteroplasmy, age, sex, and mitochondrial copy number (mtCN) with the risk of diabetes in carriers increasing by 1.62-fold (95% CI [0.97–2.7], *P* = 0.06) per 1 SD increase in T2DGRS in m.3243A>G carriers. The impact of T2DGRS on diabetes penetrance was similar to that of non-carriers (interaction *P* = 0.9). Importantly, this borderline association was maintained with a partitioned T2DGRS representing beta cell dysfunction (HR 1.1, 95% CI [1–1.2], *P* = 0.04) but not with other non-beta cell partitioned GRS (data not shown). This supports the current understanding that beta cell dysfunction is a primary cause of diabetes in m.3243A>G individuals [27].

### Most previously reported pathogenic variants are rare in a population cohort

**Figure 4:**
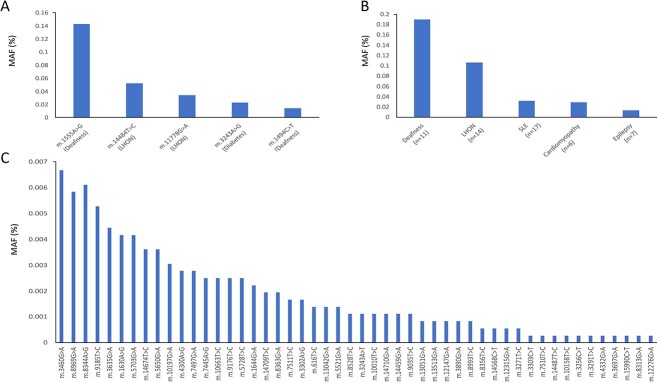
Minor allele frequency (%) of pathogenic mitochondrial variants in the UKB (A) the five most common variants (B) variants (n) grouped by disease (C) the remaining 50 variants present in the UK biobank. LHON=Leber hereditary optic neuropathy; MIDD=Maternally inherited diabetes and deafness (note: mitochondrial encephalomyopathy, lactic acidosis and stroke-like episodes (MELAS) and other phenotypes are also associated with this variant); SLE=Stroke-like episodes.

We next assessed the frequency and association of known pathogenic variants on our 15 mitochondrial-related traits. Of 73 well-characterized known pathogenic variants, 13 were not present in UKB (17.81%), 35 were very rare with a frequency of < 1:35 973 (n ≤ 5 carriers), 17 with a frequency between 1:8994–29 977 (n = 6–20 carriers), five with a frequency between 1:1799–8565 (n = 21–100 carriers; m.1494C>T, m.3243A>G, m.3460G>A, m.8344A>G, m.8969G>A), and three with a frequency > 1:1400 (m.11778G>A, n = 123 carriers; m.14484T>C, n = 190 carriers; m.1555A>G, n = 514 carriers) (Fig. 4, Table S9). Variants causing deafness were most common at 1:263 people (n = 684) followed by Leber hereditary optic neuropathy (LHON) disease-causing variants at 1:439 (n = 410) although even the move common variants showed very few affected participants, except for m.3243A>G (Table 1).

**Table 1:** Penetrance of relevant phenotype in carriers of 6 most common pathogenic variants in UKB.

Pathogenic Variant (disease)	Phenotype assessed in UKB	Phenotype frequency in carriers (%)	Phenotype frequency in non-carriers (%)
m.3243A>G (MIDD)	Diabetes	24/83 (28.9)	16 362/179 867 (0.09)
m.1555A>G (Deafness)	Hearing aid use	19/514 (3.7)	6193/179 438 (3.45)
m.14484T>C (LHON)	Bilateral visual loss	0/190	124/179 762 (0.07)
m.11778G>A (LHON)	Bilateral visual loss	0/123	124/179 829 (0.07)
m.1494C>T (Deafness)	Hearing aid use	0/53	6212/179 899 (3.45)
m.3460G>A (LHON)	Bilateral visual loss	0/24	124/179 928 (0.07)
m.8344A>G (Epilepsy)	Epilepsy	0/22	2635/179 929 (1.46)

MIDD=Maternally inherited diabetes and deafness. LHON=Leber hereditary optic neuropathy.

Most of the variants were too rare to perform statistically well-powered single variant analysis. However, for the seven variants (except m.3243A>G) where we had > 20 individuals in the EUR-like ancestry cohort, we did not observe association with the mitochondrial-related traits at a Bonferroni-corrected threshold (*P* < 0.0006) (Table S10). We also did not observe association with the respective traits when variants were aggregated by disease-associated phenotypes. The penetrance of relevant traits for these variants were very low and ranged from 0%–3.7%.

## Discussion

Our study demonstrates that large-scale WGS data offer an exciting opportunity to study the role of mitochondria in human health. This approach can identify important mitochondrial variants and provide novel insights which may lead to the possibility of reporting mitochondrial variants discovered incidentally.

Population studies allow a unique opportunity to assess the frequency of pathogenic variants which have been previously identified in cohorts referred because of presenting diseases. The m.3243A>G variant is the most common cause of adult mitochondrial disease and due to its heteroplasmic nature it is absent from whole genome data derived from genotyping arrays. Here we used WGS data to accurately genotype m.3243A>G carriers in a population cohort of ~180 000 participants and identified 83 cases with heteroplasmy ≥ 3% in blood (1 in 2167). This frequency may be an underestimate as it is well known that blood heteroplasmy reduces with age [25] and the mean age of our cohort was 56.89 years (n = 179 862; SD = 8.1, range 38.83–72.92). Previous attempts at identifying population frequency have been limited to small, and/or selected cohorts, with estimates ranging from 0.017%–1.69% [28–33].

We were surprised to find only one variant (m.3243A>G) associated with diabetes despite having large numbers of people with diabetes in our cohort. We observe that penetrance in the unselected population increased with m.3243A>G level, reaching 42% at age 50 for people with measured heteroplasmy of ≥ 10% (age adjusted heteroplasmy ~45%). The risk ratio of diabetes at ≥ 10% heteroplasmy (age-adjusted heteroplasmy ~45%) is comparable to that of pathogenic variants in well-known monogenic diabetes genes in the same cohort, as we previously published [20]. Although heteroplasmy was a major factor affecting penetrance, we also identify that polygenic risk of type 2 diabetes can also modify the penetrance. These results will need to be replicated in larger cohorts but demonstrate an exciting interplay between nuclear and mitochondrial genome variants responsible for the onset of diabetes in humans. The observation of lower penetrance in an unselected population, compared to a clinically ascertained cohort, has also been recently reported in nuclear monogenic diabetes [20] and in mitochondrial disorders such as LHON [34].

Despite the large sample size and examining 15 mitochondrial-related traits, the only trait where we have shown multiple mitochondrial variant associations is AST. We identified 12 mtDNA variants associated with AST levels, nine of which were previously reported in the same cohort when these variants were called from genotyping array data [14]. The novel lead variant m.13488C>T also showed an association with ALT, suggesting that these variants are likely to increase AST level by their effect on the liver. However, the lack of association with NAFLD and total bilirubin may also indicate the association could be driven by non-liver sources of AST such as skeletal muscle or myocardium. Additionally, the effect size on AST was small, and the lack of association with NAFLD also suggests that the high AST level was not due to hepatocellular damage/leakage but may reflect a rise in mitochondrial AST isoform rather than cytoplasmic AST [35, 36]. Mitochondrial dysfunction has been reported to play a significant role in the pathogenesis of non-alcoholic fatty liver disease (NAFLD; for review, see [37]). However, we did not identify any genome-wide significant association with NAFLD. Despite a sample size that was substantially larger, we were unable to replicate the previous borderline (*P* = 0.06) association of m.16318A>C (*P* = 0.38) [38]. The original finding may be a false-positive due to the small sample size (n ~ 300), but the disparity could also be explained by less well-defined phenotype capture in UKB and a reduced effect size in a non-clinically ascertained cohort.

Ours is the largest study of mitochondrial variants based on whole genome sequence data. This provides unique opportunity to assess the population frequency of pathogenic variants which help to better understand the burden of mitochondrial disease in population. We found that most variants were rare in the population in line with them causing rare mitochondrial disorders except for some variants causing deafness (m.1555A>G, MAF = 0.0014; m.1494C>T, MAF = 0.00014) and LHON (m.14484T>C, MAF = 0.0005; m.11778G>A, MAF = 0.0003). Both m.1555A>G and m.1494C>T cause deafness only after exposure to aminoglycoside antibiotics, which may explain why we failed to detect an association with deafness with either variants in isolation or when combined [39]. We also did not observe an association with bilateral vision loss for the LHON variants (n = 10) identified. This may be due to a combination of the lower sample size of bilateral vision loss in our cohort (n = 140) and the well-known low penetrance of the LHON variants in the unselected population [40]. Although we used mitochondrial-related traits, our phenotypes were more general and may have overshadowed the specific phenotype of mitochondrial-related diseases (such as stroke-like episodes vs. all strokes [41]). This, along with the low number of pathogenic variants and despite our large cohort, may explain the lack of association of known pathogenic variants in our study.

Our study has some limitations. Although our study was one of the largest to assess mitochondrial-wide association, we were still limited in power for non-European-like ancestry populations, specifically African-like and South Asian-like. We needed the sample size of 32 000 and 28 000 at alpha of 0.05 for variant similar to m.3243A>G to achieve the power of 0.8 for the AFR and SAS individuals, respectively*.* Similarly, it is known that the UK Biobank has a healthy volunteer selection bias which is not fully representative of the UK population [42] and may have therefore limited the inferences we can make about multisystem disease. Our stringent sample and variant filter criteria mean that it is possible that we may have excluded true causal or associated variants with these 15 mitochondrial-related traits. However, these stringent criteria allowed us to investigate the association of low level heteroplasmy. This was particularly important for m.3243A>G, which is commonly considered to be present in blood with a heteroplasmy ≥ 3% [25, 43, 44]. For our primary analysis, we used variants with heteroplasmy > 3%. Despite of our multiple stringent sample and variant level exclusion criteria to minimize false positive low heteroplasmic variant calls, we could not be certain that some of these low-level variants are false positive. We therefore also undertook sensitivity analysis at higher level of 5% and 10% heteroplasmy where the presence of false positive variants is minimal [21]. It is known that age strongly impacts blood heteroplasmy of m.3243A>G variants. We therefore use age as a covariate in the regression models with measured heteroplasmy and performed sensitivity analysis using age-corrected heteroplasmy from a published method to assess its relationship to the phenotype [25]. However, this method can overestimate the heteroplasmy at extremes of age resulting in a heteroplasmy estimate > 100%, suggesting it may over-estimate heteroplasmy for some individuals and is not necessarily generalizable to every cohort. Heteroplasmy of m.3243A>G was detected by droplet digital PCR (ddPCR) in clinically referred probands whereas heteroplasmy in the UK Biobank was from next generation sequencing data. Although these are different methods, they provide near identical heteroplasmy (Fig. S4) and is thus unlikely to affect the results of our study.

Some of the phenotypes we assessed had a lower sample size (e.g. bilateral vision loss), which will have limited our ability to robustly assess any associations and would require larger sample sizes to better perform association discovery. We also limited our analysis to single nucleotide variants and small insertions/deletions and did not assess larger deletions, which have been implicated in mitochondrial disease. Additionally, we only used heteroplasmy identified from whole blood samples, which may not reflect heteroplasmy in other tissues or organs that are more relevant to specific mitochondrial diseases.

Our study has important implications for the incidental identification of pathogenic mitochondrial variants. Diagnostic molecular genetic laboratories are moving towards using whole-genome sequencing as a first-line genetic test. This, along with a rapid rise in direct-to-consumer testing and use of WGS for research, provides an exciting opportunity to obtain information on pathogenic mitochondrial variants, well before any disease onset. We show that, when detected incidentally from blood, measured m.3243A>G heteroplasmy ≥ 10% (age-adjusted heteroplasmy ~45%) significantly increases the risk of diabetes, heart failure, deafness, and that individuals with this level or greater are more likely to experience multiple system disorders. Our findings, combined with the growing availability of prenatal testing for mitochondrial disease, indicate that reporting of m.3243A>G variant when discovered incidentally could have a significant health benefit, particularly for female individuals of reproductive age. If one decides to report this variant when found incidentally, our data suggest that this may be beneficial to individuals with age-adjusted heteroplasmy levels over 45%. However, further studies in unselected population(s) will be needed to refine this advice.

## Materials and methods

### Study subjects

#### UK biobank

The UKB is an ethically-approved population cohort of ~500 000 individuals from the UK [45]. The UKB contains deep phenotype data from self-reporting, hospital and GP records, and measurements of 30 blood biomarkers including HbA1c and liver enzymes, which are paired with detailed genetic data. Whole exome sequencing and genotyping array imputation data are available in the entire cohort, and at the time of writing (May 2023). WGS data is available on 200 030 participants of diverse genetic ancestries. The lack of selection on any specific disease phenotypes, and large sample size, makes it an ideal cohort to study rare genotype-phenotype associations. Cohort characteristics of individuals included in the current study are summarized in Table S11.

#### Clinically identified probands with m.3243A>G pathogenic variant

We included 95 probands who were referred from routine diabetes clinics in the UK to Exeter Genomics laboratory, Royal Devon University Healthcare Hospital with suspected mitochondrial-related diabetes and found to harbour the m.3243A>G variant. The study was approved by the Wales Research ethics Committee 5 (22/WA/0268). Cohort characteristics for these individuals are summarized in Table S12.

#### Clinical phenotypes

We analysed 15 diseases/traits that have been commonly associated with mitochondrial disease [1, 46] and were possible to generate from the data available in the UKB. We used self-report data, ICD9/10 codes, medication, and biomarkers to find these phenotypes (Table S7).

### Genetic data

#### Whole genome sequencing data

WGS alignment files (CRAM format) were generated by two sequencing providers, deCODE genetics and the Wellcome Trust Sanger institute, for the UK Biobank project. Briefly, genomic DNA for each sample underwent paired-end sequencing of 151 base pairs on Illumina NovaSeq6000 sequencers with the S4 flow cell (v1.0 chemistry). Data were aligned to GRCh38 before undergoing contamination and data quality control. Further detail is available in Supplementary Methods and a full detailed description is available from Supplementary Notes 1-4 in Halldorsson *et al*., [22]. We derived genetic ancestry for Europeans (EUR-like), Africans (AFR-like) and South Asians (SAS-like) via comparison of genotypes derived from the UK Biobank Axiom Array to nuclear genome principal components derived from the 1000 genomes project.

#### Mitochondrial variant calling from whole genome sequencing data

We used MitoHPC with Mutect2 in mitochondrial mode to acquire mitochondrial variants from the WGS CRAM files of the 200 030 participants [47, 48]. MitoHPC is specifically designed to detect mitochondrial single nucleotide variants (SNV) in large WGS datasets and provides accurate heteroplasmy estimates by using a consensus mitochondrial sequence for each sample. MitoHPC also provides mitochondrial copy number estimation (as a proportion of reads mapped to the mitochondrial and nuclear genomes), haplogroup determination, sequencing coverage statistics (Fig. S5), as well as quality metrics at both sample and variant levels.

We used stringent sample and variant level exclusion criteria to minimize false-positive low heteroplasmic variant calls. We excluded potentially contaminated samples (e.g. multiple dominant haplogroups in a single sample n = 736), samples with low coverage (min < 200× or mean < 500×, n = 1126), or samples with multiple nuclear DNA of mitochondrial origin (nuMT) variants flagged by MitoHPC (n = 17 485) [47]. Extremes of mitochondrial copy number (Q1-1.5×IQR and Q3 + 1.5×IQR, n = 13), were also excluded. An additional 821 samples were excluded where low quality, or missing, genotype data excluded them from the generation of a genetic relatedness matrix (n = 731) or were not able to be processed by MitoHPC (n = 90). In total, Of the 199 209 samples that were processed by MitoHPC, 179 862 (90.3%) samples passed our robust sample filtering.

Low level heteroplasmy variant calls can be false positives when called using NGS sequencing data and is impacted by the depth of sequencing coverage. Our variant level filtering was in line with previous large studies [21] and took multiple steps to minimize detection of false positive variants. Specifically, we removed low quality variants identified by GATK FilterMutectCalls as well as multiallelic indels and those identified within six known low complexity regions [21] or at 382 nuMT sites determined by MitoHPC [47]. This provided us with 15 881 variants in 179 862 individuals. Of these, 12 009 (~76%) variants had a minor allele frequency < 0.01% which would not have been reliably captured using genotyping array technology [49] and 8896 variants (56%) had minor allele count (MAC) ≥ 5. Given our stringent filtering criteria, our primary analysis used variants with ≥ 3% heteroplasmy. We also performed sensitivity analysis for variants with ≥ 5% and ≥ 10% heteroplasmy, as shown in Table 2, to remove the possible false association by low level mitochondrial heteroplasmic calls.

**Table 2:** Heteroplasmy (%) thresholds used to define heteroplasmic and homoplasmic variants.

Definition	Reference genotype	Heteroplasmic	Homoplasmic
Primary	< 3	≥ 3 and < 97	≥ 97
Sensitivity	< 5	≥ 5 and < 95	≥ 95
Sensitivity	< 10	≥ 10 and < 90	≥ 90

#### Type 2 diabetes (T2D) genetic risk score

We generated a T2D genetic risk score for individuals in the UKB based on 88 T2D associated variants from genotyping array as identified in previous genome wide association studies [50, 51] that did not include the UKB. We also generated the partitioned T2D scores as described by Udler *et al.* [52].

#### m.3243A>G testing for clinically referred probands

We used digital droplet PCR (ddPCR) on a Bio-Rad (California, USA) machine to analyse blood DNA for presence of and to determine heteroplasmy of m.3243A>G. PCR primer sequences for the m.3243A>G ddPCR assay are described by Singh *et al* [53]. Plates were run on an AutoDG Droplet Generator (Bio-Rad) with DG32 cartridges according to manufacturer’s instructions and analysed using QuantaSoft version 1.7 software (Bio-Rad). All samples were tested in triplicate and droplet data combined for final analysis of heteroplasmy. Heteroplasmy > 2% was considered positive.

#### List of known pathogenic mitochondrial variants

We reviewed all proposed pathogenic variants from the MitoMAP repository (accessed Oct 2022) https://mitomap.org/foswiki/bin/view/MITOMAP/ConfirmedMutations. For this study, we included a variant (n = 73, Table S9) if it is curated as pathogenic or likely pathogenic based on the multiple MitoMAP criteria (status = Cfrm) (https://www.mitomap.org/foswiki/bin/view/MITOMAP/MutationsCodingControlCfrm) and where available, annotated as pathogenic or likely pathogenic based on the independent criteria defined by the international ClinGen consortium e.g. https://mitomap.org/cgi-bin/search_allele?variant=7445A>G.

### Statistical analysis

#### Mitochondrial genome wide association

We performed single variant association analysis for variants with a MAC ≥ 5 (n = 8896) using REGENIE (v3.1.4) [54] for 15 selected disease/traits (Table S2). REGENIE performs genome wide association testing for large sample sizes and is robust to unbalanced case-control ratios, and controls for population structure by utilizing a genetic relatedness matrix generated from common, independent nuclear single nucleotide polymorphisms. To generate the genetic relatedness matrix, we used participants who had both a whole genome sequence and whole exome sequence data available.

For variants which were only ever heteroplasmic, or only ever homoplasmic in our cohort, we compared them against the reference genotypes. For variants where some individuals were defined as heteroplamic and some homoplasmic, our primary comparison was individuals with homoplasmic variant versus individuals with heteroplasmic or reference genotype. We performed secondary analysis by comparing heteroplasmic or homoplasmic variant genotypes together versus reference genotypes. We rank inverse normalized the continuous traits and the analysis was adjusted for age at recruitment, sex, sequencing batch, recruitment centre, mitochondrial copy number (mtCN) and the first 40 nuclear genetic principal components (PC). Our significance threshold was the Bonferroni corrected p value of < 3.75×10^−7^ calculated by number of variants and traits that were analysed (=0.05/8896/15). Power calculations were undertaken using the R package genpwr.

#### Rare variant aggregation testing

We performed aggregate testing of rare mtDNA variants (MAF < 0.1%) annotated as either missense, synonymous, or loss-of-function by Ensembl VEP [55] which were grouped by mitochondrial transcript, the d-loop, and 100 bp sliding windows at 10 bp intervals. Previously known pathogenic variants were also grouped by their known disease phenotypes (Table S9). We used REGENIE to perform burden, SKAT and ACAT aggregate tests [54] for association with all 15 traits. We used a Bonferroni corrected p value of < 2×10^−6^ as our significance threshold, calculated by number of masks and traits that were analysed (=0.05/1696/15).

#### Penetrance of diabetes for m.3243A>G

We used Kaplan-Meier survival estimates to estimate the age-dependent penetrance of diabetes. A log-rank test for equality was used to compare the penetrance of diabetes between the groups. Cox’s regression was used to compute the hazard ratio for developing diabetes with or without the adjustment of covariates. The analysis included all individuals without diabetes and individuals with diabetes without missing age at diagnosis (92% of all diabetes cases). We used STATA 16 for all analysis.

##  

*Conflict of interest statement*: The authors declare no competing interests.

## Funding

This research has been conducted using the UK Biobank Resource. This work was carried out under UK Biobank project number 9072. The current work is funded by Diabetes UK (19/0005994 and 21/0006335), MRC (MR/T00200X/1) and Wellcome Trust’s Institutional Strategic Support Fund awarded to University of Exeter. K.A.P. is funded by Wellcome Trust (219606/Z/19/Z), A.T.H. is supported by Wellcome Trust Senior Investigator award (WT098395/Z/12/Z) and S.J.P. is funded by the Wellcome Career Re-entry Fellowship (204709/Z/16/Z) and the Wellcome Centre for Mitochondrial Research (203105/Z/16/Z). The work is supported by the National Institute for Health Research (NIHR) Exeter Biomedical Research Centre, Exeter, UK. The Wellcome Trust, MRC and NIHR had no role in the design and conduct of the study; collection, management, analysis, and interpretation of the data; preparation, review, or approval of the manuscript; and decision to submit the manuscript for publication. The views expressed are those of the author(s) and not necessarily those of the Wellcome Trust, Department of Health, NHS or NIHR. For the purpose of open access, the author has applied a CC BY public copyright licence to any Author Accepted Manuscript version arising from this submission.

## Data availability

The data supporting the findings of this study are available within the article and its Supplementary Data files. Additional information for reproducing the results described in the article is available upon reasonable request and subject to a data use agreement. The UK Biobank dataset is available from https://biobank.ctsu.ox.ac.uk.

## Supplementary Material

cannon_hmg-2023-ce-00523_supplementary_methods_revision_ddad194

cannon_hmg-2023-ce-00523_supplementary_tables_revision_ddad194
