# Supplementary material for: Penetrance and expressivity of mitochondrial variants in a large clinically unselected population
Source: Hum Mol Genet. 2023 Nov 21;33(5):465–74. doi: 10.1093/hmg/ddad194 (PMC10877468; doi:10.1093/hmg/ddad194)
Supplement: cannon_hmg-2023-ce-00523_supplementary_methods_revision_ddad194 [file cannon_hmg-2023-ce-00523_supplementary_methods_revision_ddad194.docx]

**Supplementary Methods**

Full details of the sequencing methods are available in Halldorsson et al., 2022 Supplementary Notes 1-3 (1). Sequencing was performed at deCODE genetics and Wellcome Trust Sanger Institute.

**Data quality specifications**

Samples were required to have 95% of the autosome covered to a minimum of 15x as well as a minimum of 95% of mapped read pairs from the same DNA fragment with appropriate orientation and separation. Freemix sample contamination level was measured by VerifyBamID (2) whereby <1% passed, 5% failed, between 1% and 5% were further analysed with Read_haps (3). Sample discordance at non reference genotypes was required to be <3% to pass. All calculations of data quantity and coverage excluded duplicate reads, adaptors, overlapping bases from reads from the same fragment and soft clipped bases.

**Whole genome sequencing**

A normalized, target DNA concentration of 12 ng/µL in was confirmed by UV/VIS spectrophotometry and sequencing libraries were prepared using the NEBNext Ultra™ II PCR-free kit with 500ng of genomic DNA fragmented to a mean target size of 450-500 bp. End repair and A-tailing was performed in a single step followed by ligation of unique dual indexed sequencing adaptors and two rounds of SPRI-bead purification (0.6X) using an automatic 96/8- channel liquid handler (Hamilton Microlab STAR and Tecan Freedom EVO). Quality (concentration and insert size) of sequencing libraries was determined using the LabChip GX (96-samples) instrument (Perkin Elmer). Sequencing libraries were pooled using automatic 8-channel liquid handlers and sequenced using Illumina´s NovaSeq6000 instruments.

**Sequence processing pipeline**

The bcl2fastq algorithm demultiplexes the data and convert each sample into FASTQ pairs before being processed to create a CRAM file. CRAM file generation constitutes 1) Alignment to the GRCh38 reference genome 2) Fix mate pair information 3) Mark duplicate reads 4) Sort reads by genomic position.

**References**

1 Halldorsson, B.V., Eggertsson, H.P., Moore, K.H.S., Hauswedell, H., Eiriksson, O., Ulfarsson, M.O., Palsson, G., Hardarson, M.T., Oddsson, A., Jensson, B.O. et al. (2022) The sequences of 150,119 genomes in the UK Biobank. Nature, **607**, 732-740.

2 Zhang, F., Flickinger, M., Taliun, S.A.G., Abecasis, G.R., Scott, L.J., McCaroll, S.A., Pato, C.N., Boehnke, M. and Kang, H.M. (2020) Ancestry-agnostic estimation of DNA sample contamination from sequence reads. Genome Res, **30**, 185-194.

3 Eggertsson, H.P. and Halldorsson, B.V. (2021) read_haps: using read haplotypes to detect same species contamination in DNA sequences. Bioinformatics, **37**, 2215-2217.
